# Exploring the Facilitators and Barriers of Adherence to Mediterranean-Ketogenic Dietary Interventions in Parkinson’s Disease: A Qualitative Study

**DOI:** 10.1016/j.cdnut.2025.107591

**Published:** 2025-10-30

**Authors:** Kira N Tosefsky, Yolanda N Wang, Joyce ST Lam, Tamara R Cohen, Silke Appel-Cresswell

**Affiliations:** 1 Pacific Parkinson’s Research Centre, Djavad Mowafaghian Centre for Brain Health, University of British Columbia, Vancouver, BC, Canada; 2 MD/PhD Program, Faculty of Medicine, University of British Columbia, Vancouver, BC, Canada; 3 Faculty of Land and Food Systems, Food, Nutrition and Health, University of British Columbia, Vancouver, BC, Canada; 4 Faculty of Medicine, Department of Medicine (Neurology), University of British Columbia, Vancouver, BC, Canada

**Keywords:** Parkinson’s disease, Mediterranean diet, ketogenic diet, medium-chain triglycerides, behavior change intervention, qualitative analysis

## Abstract

**Background:**

Emerging research suggests indications for both Mediterranean and ketogenic diets in Parkinson’s disease (PD), with hybrid Mediterranean-ketogenic interventions potentially conferring maximal benefit. However, the acceptability of these interventions in individuals with PD (PwPs) remains to be established.

**Objectives:**

To explore and compare factors affecting PwPs’ experiences with and adherence to an 8-wk Mediterranean-ketogenic (MeDi-KD) versus an 8-wk medium-chain triglyceride (MCT)-supplemented Mediterranean (MeDi-MCT) diet.

**Methods:**

Forty-eight PwPs took part in ≥1 phase of the crossover trial. Sixty-seven semistructured interviews were completed by 44 participants after each intervention. Interview questions, structured using the Theory of Planned Behavior (TPB), explored how participant attitudes, control beliefs, environmental contexts, and social influences impacted study diet adherence. Reflexive thematic analysis was applied to interview transcripts.

**Results:**

Two subthemes each emerged under of the overall themes of “Attitudes,” “Perceived behavioral control,” and “Subjective norms” within the TPB framework. Attitudes toward MeDi-KD and MeDi-MCT compositions were polarized, based on varying levels of familiarity with similar dietary patterns, diverging taste preferences, and wide-ranging expectations of effects on PD-specific and general health outcomes. Capability and resources grouped together naturally under the “Perceived behavioral control” theme. Within this domain, competing personal responsibilities, attentional resources diverted to disease management tasks, PD-related apathy, and motor deficits compromising cooking skills, all proved especially problematic during the labor-intensive MeDi-KD phase. In the “Subjective norms” domain, household and cultural acceptance of study diets proved crucial for sustained adherence. Our study’s registered dietitian played a pivotal role in supporting adherence through the direct provision of informational, instrumental, and social support, as well as by engaging care partners to bolster social support within the home.

**Conclusions:**

Our qualitative analysis illuminates behavioral and contextual determinants of adherence to keto-adapted MeDis in PwPs, informing more sustainable dietary implementation strategies relevant both to future trial design and dietetics clinical practice.

This trial was registered at clinicaltrials.gov as NCT05469997.

## Introduction

As of 2021, nearly 12 million individuals were diagnosed with Parkinson’s disease (PD) worldwide, with this number projected to reach 17 million by 2035 [1]. In addition to standard pharmacologic and device-aided therapies, including deep brain stimulation, levodopa-carbidopa intestinal gel infusion (LCIG), and subcutaneous infusion of the dopamine agonist, apomorphine [2,3], maintenance of adequate nutrition is essential for optimizing quality of life in individuals with PD (PwPs) [[3], [4], [5], [6]]. A growing body of evidence further supports a potential role for specific dietary interventions in modifying disease risk and/or its course [7,8]. Observational studies have identified significant inverse associations between the Mediterranean (MeDi), Mediterranean-Dietary Approaches to Stop Hypertension Intervention for Neurodegenerative Delay, plant-based, and generally high-quality diets (as defined by indices such as the Alternative Healthy Eating Index) with PD incidence [[9], [10], [11], [12], [13], [14], [15]], age of onset [16], symptom severity [17,18], disease progression [14,19], and all-cause mortality [20]. Additionally, 8 pilot randomized controlled trials of ketogenic and Mediterranean-dietary interventions in PwPs have reported variable improvements in motor, nonmotor, and cognitive symptoms [[21], [22], [23], [24], [25], [26], [27], [28]]. Preliminary findings from these early-phase studies warrant further investigation in larger-scale phase III efficacy trials; however, methodological issues such as poor dietary adherence and study retention rates make these studies particularly challenging to execute [29,30].

Qualitative research can offer key insights into the individual, interventional, and environmental-level factors that may impact dietary adherence in populations of interest [31,32]. Select studies have, thus far, examined the diet-related experiences of PwPs from a qualitative perspective [[33], [34], [35], [36], [37], [38]], primarily in an observational, real-world setting. These real-world studies have identified common barriers to healthy diet adherence in PwPs, including coping with cravings, eating in social settings, challenges with food preparation, and limited knowledge regarding food-medication interactions [33,35,36]. Both PwPs and their care partners report changes in taste, smell, and appetite, management of food-medication interactions, swallowing, and gastrointestinal issues as key PD-associated concerns affecting dietary choices and food intake [35,36]. PwPs repeatedly echo a desire for better counseling regarding the best dietary regimen for those with the disease, with many turning to online discussion forums in the absence of clear clinician guidance [33].

In response to this evident interest in dietary self-management strategies in the PD community, several interventional studies of Mediterranean (MeDi) [25], and ketogenic (KD) diets have been conducted, and have provided preliminary evidence for potential symptomatic benefits [[21], [22], [23], [24],^27^,^34^,^39^,^40^]. However, only one of these conducted a qualitative assessment of participant experiences using a phenomenological approach, to examine the impact of a standard KD on quality of life of PwP in the setting of a nonrandomized, pilot longitudinal study [34]. PwP in this study detailed quality of life improvements related to their study involvement, emphasizing the sense of empowerment that can stem from nutritional self-management of their chronic disease [34]. However, the 7 participants interviewed constituted a subset of a non-randomized sample who voluntarily elected to continue a KD after participating in a 12-wk randomized trial [22]. As such, the generalizability of these findings to randomly selected PD cohorts, and to those without prior experience with the KD, remains to be investigated. Factors affecting PwPs experiences with and adherence to MeDi diets, or a combined MeDi-KD intervention, has yet to be investigated.

To address this gap in the literature, we conducted a phase II, random-order crossover trial of 2 combined Mediterranean-ketogenic interventions in PwP (KIM Trial): *1*) a high-fat, low-carbohydrate Mediterranean diet (MeDi-KD) and *2*) a non–carbohydrate-restricted Mediterranean diet supplemented with ketogenic medium-chain triglyceride oil (MeDi-MCT).

The primary aims of the trial were to assess the safety and feasibility of these 2 diets in PwPs as plausibly healthier and more feasible alternatives to standard KDs [41,42]. The main results of this trial are reported in a separate publication [43] and revealed substantial remaining adherence challenges, despite being ostensibly more flexible than standard KD regimens [41,44]. The study presented herein aimed to further probe PwPs using semistructured interviews to explore the barriers and facilitators of adherence to the MeDi-KD and MeDi-MCT interventions. By illuminating experiential determinants of dietary adherence in a research context, our study directly informs the design of future phase III dietary intervention studies in PD and offers practical insights for the ultimate implementation of such regimens in real-world dietetics practice.

## Methods

### Trial design

The Mediterranean-Ketogenic Dietary Interventions in Parkinson’s Disease (KIM) Trial (NCT05469997) was approved by the UBC Clinical Research Ethics Board (H21-03747) and was performed between April 2023 and October 2024. Complete trial methods were described previously [43]. In brief, patients with mild-to-moderate PD on stable (including 0 mg) doses of anti-Parkinsonian medication were recruited from the patient database of a tertiary care Movement Disorders Clinic in Vancouver, BC, Canada, or through self-referral.

Consenting participants engaged in two 8-wk interventions—the MeDi-KD and MeDi-MCT—separated by an 8-wk washout. The MeDi-KD intervention involved restriction of carbohydrates to 4% to 10% of daily calories; the remaining calories were derived from lean proteins (10%–20%) and fats (69%–82%), with a focus on unsaturated fat sources, in keeping with MeDi principles [45]. The MeDi-MCT diet involved supplementation of a non–carbohydrate-restricted MeDi with ≤20 mL MCT twice daily (Nutiva MCT oil; Nutiva Inc; NPN: 80086912). The MeDi component of the MeDi-MCT involved consumption of food groups proportions based on Canada’s Food Guide (½ plate composed of fruit and vegetables, ¼ protein, and ¼ whole grain), emphasizing key MeDi protein and fat sources such as fatty fish, olive oil, nuts and legumes. Summary descriptions provided to participants for each of these 2 diets are included in Supplemental Data File 2.

Participants attended in-person study visits at the beginning and end of each intervention phase, wherein they completed clinical assessments with the study coordinator (KNT) or other qualified research personnel and met with the study’s registered dietitian (YNW). All other intervention components, including weekly 30 min check-in calls with YNW, were completed remotely, and participants were provided with online access to recipes via the website, KetoDietCalculator (https://www.ketodietcalculator.org/about/). Participants were asked to record 1-d food intake in food diaries as well as to test capillary ketone levels using an at-home monitoring device on a once-weekly basis. Participants were compensated for travel to study visits. All participants were invited to participate in semistructured interviews at the end of the 8-wk interventions.

### Interviews

The interview script was developed based on the principles of the Theory of Planned Behavior (TPB; Table 1) [46]. The TPB is a well-utilized theory in dietary research with constructs addressing attitudinal, self-regulatory, environmental, and social factors affecting behavioral initiation and maintenance [[47], [48], [49], [50], [51]]. Several lifestyle intervention studies have demonstrated its capacity to predict health behavior maintenance for ≤12 wk [49,52,53].

**TABLE 1:** Interview script mapped to Theory of Planned Behavior constructs.

Domain	Question
Attitudes/experiences	1. What did you like about the MeDi-MCT/MeDi-KD^1^ intervention? 2. What did you dislike about the MeDi-MCT/MeDi-KD^1^ intervention? 3. How did you feel about having to take the MCT oil supplement twice daily? 4. How did you feel about having to measure your Ketone levels every week? 5. How did you feel about having to record your 1-day food intake once per week?
Subjective norms	How similar or different were the dietary interventions in this trial from your day-to-day diet before the study?
Perceived behavioral control	1. How would you rate the overall feasibility of the dietary approach in daily life? a. No problem at all, doing it every single day b. No real problem but I skip several times a month c. Difficult, I skip >1 time a week d. Very difficult, I can barely execute it 2. Have you had any breaks in between your diet? a. No b. Yes – Due to practical difficulty c. Yes – Due to side effects of the diet d. Yes – Due to other health issues 3. What were the barriers associated with adhering to these dietary interventions? 4. How long did it take you, if ever, to get used to the new dietary regimens?
Behavioral intentions	1. How likely are you to adhere to the MeDi-MCT/MeDi-KD diets (and each component individually) in the future? Rate from 1 to 5: 1 – Not likely at all; 5 – Will adhere to the diet/practice for sure. 2. If you could change anything about the MeDi-MCT/MeDi-KD, what would it be?

Abbreviations: KD, ketone diet; MCT, medium-chain triglyceride; MeDi, Mediterranean diet.

1 Mention of the MeDi-MCT compared with MeDi-KD in the question depended on which intervention participants were completing (or withdrawing from) at the time of the interview.

Ranking questions querying attitudes to various components of the interventions, subjective norms, facets of perceived behavioral control, and future behavioral intentions were employed as prompts for open-ended discussion between the participant and interviewer. Interviews were conducted at in-person study visits, primarily by the study dietitian, YNW, who had established longitudinal relationships with participants, starting at their first study visits and developed through weekly follow-up phone calls. In cases in which YNW’s availability did not allow her participation, interviews were conducted by the primary study coordinator, KNT, or trained research assistants, each of whom were familiar to the participants from baseline visits and remote scheduling communications. Interviews were conducted in private clinic rooms, with care partners present if preferred by the participant, and audio recorded with explicit permission. Structured interview questions directly probed participants’ experiences related to the composition of each dietary intervention, as well as with tasks such as once-weekly ketone self-monitoring and once-weekly recording of single-day food intake.

Due to study attrition, we anticipated that some participants would provide feedback for both interventions, whereas others would only have experience with and therefore provide feedback on a single intervention. For consistency, each interview transcript was treated as a discrete unit of data and coded using the same analytic framework, with themes developed across the dataset, rather than weighted by the number of interviews per individual. Participants who completed 2 interviews enriched the analysis by providing longitudinal perspectives and opportunities for between-intervention comparison, whereas interviews from participants who withdrew from the study provided insight into the unique experiences of individuals who deemed their individual adherence barriers insurmountable. In addition to the questions provided to intervention completers, participants interviewed at the time of intervention withdrawal were asked additional questions about their reasons for withdrawal.

### Data analysis

Interviews were transcribed using the automated transcription software in NVivo 14, with manual editing for accuracy and cleaning for legibility by KNT. Reflexive thematic analysis of interview transcripts was conducted in NVivo 14 according to Braune and Clarke’s 6-phase method [54]. Data familiarization was conducted for all interview transcripts by the KIM trial primary study coordinator, KNT, and by YNW for a randomly selected subset of transcripts.

Initial inductive coding was largely descriptive and evolved to be more interpretive in nature throughout the coding process [54,55]. Coding was conducted by KNT and YNW independently after data familiarization, with each researcher engaging in reflexivity throughout the coding process by maintaining a log of memos detailing text interpretations, rationales for grouping decisions, and reflection on how these judgments were informed by their distinct academic backgrounds and relationships with participants. After data familiarization and initial code generation, KNT and YNW met to compare codes, discuss interpretative differences documented in memos, and finalize the coding strategy. Overall similarity between the initial codes generated by KNT upon review of the full dataset and YNW upon review of the partial dataset provided reassurance that saturation had been achieved [56]. After this peer debriefing, KNT applied the consensus coding approach in the third step of Braune and Clarke’s method—interpreting and grouping codes into candidate subthemes [54] from which representative quotes have been selected for presentation. The number of subthemes was determined in a data-driven manner to optimally capture the diversity of participant experiences while summarizing recurring patterns and key concepts in a coherent manner. The codebook and candidate subthemes were reviewed with YNW, TRC, and SA-C. KNT then incorporated this feedback—merging, splitting, discarding, and renaming themes to concisely represent expressed participant experiences and address our key research questions reagarding barriers and facilitators of adherence [57]. Subthemes were subsequently grouped under deductively applied themes derived from the TPB constructs. A final codebook was circulated to and approved by YNW, TRC, and SA-C.

## Results

### Participant characteristics

Of the 52 participants randomized, 48 started 1 of the 2 interventions, 41 participants completed ≥1 intervention, and 33 completed both intervention phases. Twenty-three participants completed an interview after both intervention phases, and 21 participants completed an interview after 1 intervention phase, for a total of 44 interview participants and 67 completed interviews. Sixty-two interviews were conducted at the time of intervention completion and 5 at the time of withdrawal. Twelve participants declined to be interviewed at the time of intervention completion, and 17 declined at the time of intervention withdrawal. Aggregate participant characteristics are provided in Table 2 [[58], [59], [60], [61]]. Individual participant characteristics are provided in Supplemental Data File 1; Supplemental Table 1. The demographics of our interviewees were representative of those of our full study cohort [43]. In brief, the median age of interviewees was 68 y (IQR: 62, 74 y) with a median disease duration of 8 y (IQR: 4, 10 y) and mild-to-moderate disease (Hoehn & Yahr stages I–III) as per our inclusion criteria. Thirteen (30%) participants were female, 39 (89%) Caucasian, 33 (79%) had a live-in partner, and 9 (21%) were employed at the time of study enrolment, with a median of 18 y (IQR: 16, 20 y) of education. Diets at study intake were generally not aligned with MeDi principles, with a median baseline Mediterranean Diet Adherence Screener (MEDAS) score of 5 (IQR: 4, 6) of a maximum of 14 [58,62]. As per our exclusion criteria, participants were not following a KD or taking an MCT-based supplement at baseline.

**TABLE 2:** Summary of participant characteristics.

Characteristic	*n* = 44^1^
Disease duration, y	8 (4, 10)
Age, y	68 (62, 74)
Sex	
Female	13 (30%)
Male	31 (70%)
Ethnicity	
Caucasian	39 (89%)
East Asian	4 (9.1%)
South Asian	1 (2.3%)
Years education	18 (16, 20)
BMI, kg/m^2^	25.8 (22.3, 29.0)
Hoehn & Yahr Stage	
0	0 (0%)
I	7 (16%)
II	34 (77%)
III	3 (6.8%)
Employed	9 (21%)
Partnered	33 (79%)
Married	30 (70%)
MEDAS^2^ Score	5.00 (4.00, 6.00)
MDS-UPDRS^3^ Part I	10.0 (6.0, 12.5)
MDS-UPDRS^3^ Part II	8.0 (5.5, 11.0)
MDS-UPDRS^3^ Part III	25 (17, 35)
MDS-UPDRS^3^ Part IV	4.0 (0.0, 6.5)
MoCA^4^ Total Score	27.00 (26.00, 29.00)
PD-CRS^5^ Total Score	92 (72, 106)
Constipation	11 (25%)

Abbreviations: BMI, body mass index; MDS-UPDRS, Movement Disorder Society-Unified Parkinson’s Disease Rating Scale; MEDAS, Mediterranean Diet Adherence Score; MoCA, Montreal Cognitive Assessment; PD-CRS, Parkinson’s Disease Cognitive Rating Scale.

1 Data reported as median (Q1, Q3) or *n* (%).

2 Mediterranean Diet Adherence Score [58].

3 Movement Disorder Society-Unified Parkinson’s Disease Rating Scale, part III assessed in the “ON” state [59].

4 Montreal Cognitive Assessment [60].

5 Parkinson’s Disease Cognitive Rating Scale [61].

**TABLE 3:** Themes, subthemes, and codes identified from qualitative analysis of participant interviews.

Theme	Subtheme	Code	
		Facilitator	Barrier
Attitudes	Diet composition	• Familiarity with study diet • Meal plans were palatable • Diet perceived as flexible • Positive attitude toward dietary change	• Challenge adapting to unfamiliar foods • Meal plans or MCT oil were unpalatable • Diet perceived as too restrictive
	Expected outcomes	• Diet perceived as generally healthy • Experienced symptom improvement	• Diet perceived as generally unhealthy • Experienced adverse side effects • Perceived lack of evidence for health benefit • Lack of experienced symptomatic benefits
Perceived behavioral control	Capability	• Versatile cooking skills • High trait self-efficacy • Food diaries increased adherence self-efficacy • Ketone readings reinforced efforts	• Inadequate food preparation skills • Food diaries were onerous • Low ketone readings discouraged efforts • Ketone self-measurement was technically challenging
	Resources	• Recipe ingredients were readily accessible • MCT doses were easy to integrate into routine	• Recipe ingredients were hard to find • Grocery lists were more expensive than usual • Competing disease management tasks • Following diet instructions was attentionally taxing • MCT doses were difficult to integrate into routine • Time-intensive food preparation
Subjective norms	Household, cultural, and community norms	• Tangible support and encouragement from care partner	• Diets incompatible with household or community cultural cuisine • Diets viewed as incompatible with eating norms outside the home
	Professional support	• Information, implementation strategies and encouragement from RD	

Abbreviations: MCT, medium-chain triglyceride; RD, registered dietician.

Most interviews lasted between 15 and 30 min, although they continued for ≤1 h in select cases in which participants wished to elaborate further on their experiences. Although interviews were shorter than the typical duration of in-depth semistructured interviews [63], this reflected the study context rather than limited data generation. Participants had multiple prior opportunities to reflect on and discuss their experiences during check-in sessions with the study dietitian and the study visit preceding the interview. Consequently, participants had already established rapport with the interviewer and were typically well-prepared to provide detailed feedback, and interviewers, in turn, were familiar with the participants’ experiences, enabling efficient and substantive discussions in an abbreviated timeframe.

Within the 3 themes defined by the TPB constructs, 2 subthemes were identified in relation to ‘Attitudes’ (‘Diet compositions’ and ‘Expected outcomes’), 6 in ‘Perceived behavioral control’ (‘Self-monitoring feedback,’ ‘Self-efficacy,’ ‘Intervention-related appetite changes,’ ‘Time and attentional resources,’ ‘Food preparation skills,’ and ‘Food cost and access’) and 2 in ‘Subjective norms’ (‘Household norms and cultural factors’ and ‘Social influences outside the home’) (Table 3).

### Theme 1: Attitudes

#### Attitudes, subtheme 1: Diet compositions

Media-related aspects common to both diet phases were generally received favorably, with many participants divulging a familiarity with and positive attitudes toward the MeDis at the time of enrolment:

“We’re used to kind of we enjoy the Mediterranean diet stuff and we enjoy Greek food and the pastas. We enjoy the salads and, you know, the oils.” (K067; male, age 81 y, disease duration 8 y, not working, partnered, MeDi-KD)

One aspect of the MeDi with which several participants reported being less accustomed to was its emphasis on pulses and legumes:

“I found it hard to incorporate beans and legumes in the diet.” (K009; female, age 76 y, disease duration 6 y, not working, partnered, MeDi-MCT)

Others expressed a positive attitude toward modification of their baseline diets, reflecting beliefs that the study diets represented a healthier alternative:

“I see it as a good move to help healthier eating.” (K022; male, age 80 y, disease duration 10 y, working part-time, partnered, MeDi-MCT)

“I felt like I was eating healthier than my previous diet.” (K017; female, age 69 y, disease duration 2 y, not working, partnered, MeDi-KD)

As no participants had previously attempted to combine MeDis with ketogenic regimens, their perspectives regarding the palatability of the MeDi-KD compared with the MeDi-MCT were primarily shaped by their trial experiences. Thirteen (41%) and 13 (37%) participants commented favorably on the palatability of the MeDi-KD and MeDi-MCT, respectively, compared with 7 (22%) and 9 (26%) conveying distaste for each of these meal plans (Supplemental Data File 1; Supplemental Table 2). The exaggerated emphasis on olive oil in the MeDi-KD was the most prominent source of mixed reviews regarding its palatability, although the texture and flavor of the MCT oil itself was the most divisive issue concerning the palatability of the MeDi-MCT.

“I’ve never used so much olive oil in my life…but I’ve really lost my sense of taste and smell. So I don’t taste it, but it creates a greasy feeling in your mouth.” (K059; male, age 74 y, disease duration 3 y, not working, partnered, MeDi-KD)

“The MCT oil. It tasted like - it reminded me of motor oil. It wasn’t as flavorful as olive oil. And it I just didn’t like it.” (K039; male, age 68 y, disease duration 3 y, not working, partnered, MeDi-MCT)

More consistent than any advantage of the MeDi-KD compared with the MeDi-MCT in terms of palatability was the advantage seen in the greater flexibility of the MeDi-MCT:

“I think overall I felt less inhibited by the circumstances of this diet itself…The MCT one is more flexible.” (K048; male, age 56 y, disease duration 19 y, working part-time, partnered, MeDi-MCT)

“My diet is usually quite vegetable heavy, and I found that because of the requirements for the Keto diet and restricting the amount of fruits and vegetables in terms of volume, that that was more restrictive.” (K049, male, age 60 y, disease duration <1 y, working part-time, partnered, MeDi-KD)

#### Attitudes, subtheme 2: Expected outcomes

As alluded to by some participants, the MeDi-KD’s restriction of nutritious foods such as fruits, vegetables, and whole grains, in conjunction with its high-fat content, inspired 11 (34%) participants to cast doubts about its healthfulness, particularly among those linking healthful eating with weight management (Supplemental Data File 1; Supplemental Table 2):

“It’s an intellectual thing, that maybe I shouldn’t be eating that much fat.” (K063; male, age 61 y, disease duration 4 y, working full-time, partnered, MeDi-KD).

Naturally, participants who instead viewed the MeDi-KD (15 [47%]) or MeDi-MCT (9 [26%]) (Supplemental Data File 1; Supplemental Table 2) as “clean” or “healthy” were more likely to entertain incorporating aspects of these regimens into their diets beyond completion of the study, even if strict long-term adherence was felt to be too demanding. Participants frequently noted reductions in highly processed food and sweet intake as specific healthful aspects of both interventions worth continuing:

“I feel like I want to continue eating more fish. I probably will try to eat less sugar. Because I think there’s something there… I think trying to have good fats. Olive oil, avocado. Try to keep focusing on that. And avoiding simple carbs, for sure.” (K060; female, age 54 y, disease duration 1 y, not working, partnered, MeDi-KD)

“I’ll probably stay on the Mediterranean one like. Or sort of the Mediterranean-esque sort of, you know, that concept. I like that because it’s cleaner.” (K019; female, age 75 y, disease duration 12 y, not working, single, MeDi-MCT)

Although some participants found current suggestions of PD-specific benefits of ketogenic interventions sufficiently compelling to maintain ongoing adherence, others were notably more conservative. At the time of recruitment and consent, study team members carefully emphasized that neither of the study diets had, to date, been proven effective for modifying disease progression or alleviating PD symptoms. Understanding this message, participants explained that their likelihood of adhering to the MeDi-KD (4, 12%) or MeDi-MCT (13, 37%) in the future would depend on the emergence of further evidence for their therapeutic role in PD (Supplemental Data File 1; Supplemental Table 2):

“If there was evidence to support it for Parkinson’s, it wouldn’t be impossible [...] If there was evidence to support ketosis, I would do whatever I had to do.” (K005; female, age 63 y, disease duration 22 y, not working, partnered, MeDi-KD)

“If I firmly believe that there’s a benefit with the MCT, I’ll continue with it. But I’d like to see a study that shows that the MCT oil has an impact.” (K035; male, age 72 y, disease duration 6 y, working part-time, partnered, MeDi-MCT)

Attitudes toward each diet further evolved throughout the trial in response to perceived physical, cognitive, mood, and functional changes. Of these, weight loss (10, 23% of interviewees) and constipation relief (14, 32% of interviewees) proved to be the most salient reinforcers of dietary adherence, while reduced energy levels (3, 9%) and exercise capacity (4, 13%) deterred full commitment to the MeDi-KD, specifically:

“I lost weight, so that was encouraging, so that actually helped me stay motivated to try.” (K048; male, age 56 y, disease duration 19 y, working part-time, partnered, MeDi-KD)

“[The MCT oil] had the positive impact that I described earlier, of easing constipation. So that makes me want to say to myself, I’ve got to find what’s the right amount to continue, because prior to that, I was having serious difficulties with it.” (K022; male, age 80 y, disease duration 10 y, working part-time, partnered, MeDi-MCT)

“I was more tired when I was doing physical activity, especially.” (K053; female, age 56 y, disease duration 10 y, not working, partnered, MeDi-KD)

Among participants who withdrew from the study, more pronounced adverse physical side effects were the most common proximate cause of shifts in attitudes toward, and ultimately willingness to proceed with, the study diets. For 2 (6%) participants, the MCT oil precipitated intolerable gastrointestinal side effects, leading to study withdrawal:

“It was particularly in the mornings. It triggered bowel movements. So much so that I found that if I couldn’t plan to go out in the morning because I needed to be close to a bathroom until at least midday.” (K006; female, age 68 y, disease duration 8 y, not working, single, MeDi-MCT, ∗withdrew from MeDi-MCT)

“It made me nauseous, and I had quite a bit of stomach cramping.” (K046; female, age 63 y, disease duration 5 y, not working, single, MeDi-MCT, ∗withdrew from MeDi-MCT)

In another case leading to withdrawal, the high-fat content of the MeDi-KD resulted in a substantial apparent delay of gastric emptying and therefore levodopa absorption, profoundly extending “OFF” time:

“My Parkinson’s symptoms (such as tremor, balance, general muscular coordination; mental and emotional acuity) started to deteriorate and become increasingly compromised. Matters reached a point over the weekend where I realized that what I was experiencing was the effects of insufficient dopamine levels arising out of a failure of sufficient Sinemet to be taken up by the receptors in my gut. I reached this conclusion when I realized that my stomach was taking 10 to 12 hours after a high-fat meal to clear (which was unsustainable just from a digestion perspective); and, that this had the effect of blocking the delivery of sufficient CD/LD [carbidopa/levodopa, a common combined medication to manage the symptoms of PD] to the receptors in my gut to provide an adequate dosage to manage my Parkinson symptoms.” (K014; male, age 63 y, disease duration 6 y, not working, single, MeDi-KD, written comment, ∗withdrew from MeDi-KD)

### Theme 2: Perceived behavioral control

#### Perceived behavioral control, subtheme 2: Capability

Among participants for whom intervention side effects were absent or tolerable, high trait self-efficacy distinguished a subset of participants highly committed to the trial interventions:

“It was just willpower…If I agreed to do this study, then I need to do it properly.” (K038; female, age 75 y, disease duration 2 y, not working, partnered, MeDi-KD)

“As long as someone has the discipline to do it, it’s not hard. It just requires the discipline to do it for sure.” (K055; male, age 79 y, disease duration 5 y, not working, partnered, MeDi-KD)

Biofeedback in the form of ketone self-monitoring was credited for bolstering adherence self-efficacy in 9 (28%) and 5 (14%) participants during the MeDi-KD and MCT phases, respectively (Supplemental Data File 1; Supplemental Table 2):

“I wanted to know that my ketone levels were where they were supposed to be, you know, and sometimes they weren’t. So I’d work extra hard to make them better. Yeah. And I’m just curious.” (K056; female, age 64 y, disease duration 12 y, not working, partnered, MeDi-KD).

However, many participants struggled to sustain non-trace ketone readings despite high self-reported levels of adherence to both diets. Across the 2 interventions, 7 (10%) references were made to low ketone readings discouraging study diet adherence (Supplemental Data File 1; Supplemental Table 2):

“I was disappointed that I wasn’t able to get the count up above 0.2...I felt like I was failing the study.” (K020; male, age 76 y, disease duration 5 y, not working, single, MeDi-KD)

In contrast, 7 (16%) interviewees remarked on the helpfulness of weekly recording of 24-h food intake, and none commented on this practice compromising their adherence capacity or motivation (Supplemental Data File 1; Supplemental Table 2):

“It made me pay more attention obviously to what I was eating. And so that was good. I thought that was very worthwhile.” (K017; female, age 69 y, disease duration 2 y, not working, partnered, MeDi-KD).

“I think that doing the weekly diarizing meant that I was paying attention. Without having that requirement, I think it would be more difficult to pay attention to it as much as I did.” (K049; male, age 60 y, disease duration <1 y, working part-time, partnered, MeDi-KD)

Whereas 14 (21%) of participants found ketone self-monitoring procedures burdensome or technically challenging, only 3 (4%) made similar comments in reference to the keeping of food diaries (Supplemental Data File 1; Supplemental Table 2). K049 clarified that, while weekly recording of intake was helpful and reasonable, keeping a food diary,

“Would be onerous to do more often than once a week. Like writing it out daily, for example.” (K049; male, age 60 y, disease duration <1 y, working part-time, partnered, MeDi-KD)

Other interviewees related difficulties with intervention execution less to self-regulation around eating behaviors and more to inadequate food preparation skills, often due to PD motor symptoms:

“Maybe just feeling like some slowness and other things that makes it more difficult during the actual physical preparing of the food.” (K062; male, age 77 y, disease duration 9 y, not working, partnered, MeDi-MCT)

#### Perceived behavioral control, subtheme 2: Resources

Separate from individual confidence and skills informing the “Capability” subtheme, other participants attributed suboptimal adherence to lifestyle and disease-related circumstantial constraints. Work obligations and complex medication schedules limited participants’ residual time and attention to devote to the interventions, with PD-related apathy aggravating the sense of overwhelm from these responsibilities. The competing demand for time-intensive food preparation proved more problematic during the MeDi-KD (21, 66%) than the MeDi-MCT (8, 23%) phase (Supplemental Data File 1; Supplemental Table 2), due to its added requirement of weighing out ingredients to meet macronutrient targets and relative inaccessibility of compatible ready-made meal and snack options:

“I guess I have a certain amount of apathy. I mean, that’s part of the problem. I think that it’s difficult for me to be diligent on making sure that everything is recorded. There is a diary, and I’m weighing things, and that sort of thing…but I’m trying to really just keep going with my meds and everything. So if I have too much to concentrate on, it’s hard.” (K016; male, age 59 y, disease duration 13 y, not working, single, MeDi-KD, ∗withdrew from MeDi-KD)

“What it basically did is made me think of nothing but food and pills all day long.” (K056; female, age 64 y, disease duration 12 y, not working, partnered, MeDi-KD)

“I’m still working and so I cannot really dedicate my whole day to this intervention.” (K025; male, age 50 y, disease duration 9 y, working, partnered, MeDi-KD)

In contrast to the MeDi-KD’s intensive food preparation tasks, a minority of participants (9, 26%) described the MeDi-MCT’s unique task of twice-daily supplement administration (morning and evening) as intrusive:

“I’m used to taking pills. So I just work it into my schedule. You know, as long as I’m having a meal, I’ll remember to take the supplement.” (K032; male, age 67 y, disease duration 12 y, not working, partnered, MeDi-MCT)

Food access and accessibility issues were likewise commented on most frequently in relation to expensive, specialty “keto-friendly” ingredients during the MeDi-KD phase 13 (41%), particularly by those living rurally (*n* = 4, 9%) and those with smaller food budgets:

“I’m on a limited income, and its pricier to eat this way.” (K016; male, age 59 y, disease duration 13 y, not working, single, MeDi-KD, ∗withdrew from MeDi-KD)

“The keto diet took it to the next level of expense. It’s just a lot of those items. They’re not something the average person is going to use in their daily life. You know, like the almond flour and coconut flour or whatever. And all these things are all very expensive.” (K053; female, age 56 y, disease duration 10 y, not working, partnered, MeDi-KD)

“I think is it’s also…we live on an island. Even coming into shop, it’s a ridiculously expensive diet. Eating avocados, that protein powder...probably all groceries went up. It’s hard to gauge, but it was very expensive. And even trying to find the protein powder was hard.” (K056; female, age 64 y, disease duration 12 y, not working, partnered, MeDi-KD)

Though cost and access were most commonly raised as issues in relation to the MeDi-KD, recommendations for high-cost produce in the MeDi-MCT elicited similar feedback:

“I think the cost of food - avocadoes, those kind of things - have been kicked up. Some things are getting hard to find - so maybe, maybe some alternatives that they people could use as an experiment would be useful.” (K051; male, age 68 y, disease duration 8 y, not working, partnered, MeDi-MCT)

#### Subjective norms

##### Subjective norms, subtheme 1: Household and community norms

Resource constraints were exacerbated in several cases by the practical realities of participants’ social contexts, posing obstacles to sustained diet adherence. For example, familial aversion to the study diets often left participants preparing multiple separate dishes for family meals, heightening the time and expense associated with meal preparation:

“I had to make one meal for my kids, and one for my wife and me differently.” (K034; male, age 57 y, disease duration 2 y, not working, partnered, MeDi-KD)

“My wife is a pescatarian and…Just the aspect of trying to blend a pescatarian or vegetarian diet with this one was not easy. I think it would have been a lot easier if I was just by myself.” (K008; male, age 71 y, disease duration 10 y, not working, partnered, MeDi-KD)

Several ethnic minority participants attributed conflicts between study diets and traditional cuisines consumed within the home as the principal barrier to familial acceptance of the trial regimens. For example, restrictions on rice intake during the MeDi-KD proved particularly problematic for participants whose families were accustomed to traditional Asian cuisines:

“I’m Korean and the Korean diet usually has a big bowl of rice usually and then all the side dishes and entrees, they are designed to actually have this, you know, rice. So, you know, keto diet is very different.” (K025; male, age 50 y, disease duration 9 y, working part-time, partnered, MeDi-KD)

Conversely, many participants’ family members played essential roles in offsetting individual limitations in food preparation capacity, with care partners often serving as households’ primary cooks. This arrangement was more common among male than female participants:

“[My wife] would just automatically cook it…She found it very easy.” (K032; male, age 67 y, disease duration 12 y, not working, partnered, MeDi-MCT)

“Mostly my wife was the one to do the cooking. And she had to change her style.” (K066; male, age 78 y, disease duration 4 y, not working, partnered, MeDi-MCT)

“[My wife] cooked my meals every single day. She’s pretty adaptable to these kind of things, and she likes cooking.” (K027; male, age 70 y, disease duration 8 y, not working, partnered, MeDi-KD)

Only 1 woman in our study described a spouse as primarily responsible for the household cooking, and—in contrast to the men in such a position—found this setup to be an adherence barrier due to conflicts between the study diet principles and her husband’s preferred cooking style:

“I wasn’t the main person preparing most of the food - it was my husband. And he does more Chinese cooking. So I think if somebody else had prepared the keto foods for me, I would have been okay. I would have done better.” (K033; female, age 64 y, disease duration 7 y, not working, partnered, MeDi-KD)

Even among participants who were able to integrate the dietary programs into their household routines, social eating occasions outside the home represented major adherence obstacles during both the MeDi-KD (16, 50%) and MeDi-MCT (12, 34%) (Supplemental Data File 1; Supplemental Table 2). Participant responses to perceived social norms varied: some declined invitations to eat in settings where their diet-compatible food choices would be considered unusual, others attempted to make diet-compatible selections despite social pressures, whereas others made exceptions to strict dietary adherence for social occasions:

“So an example would be we got invited to my daughter’s for dinner the other night. Well she was having lasagna. So I said no thank you.” (K022; male, age 80 y, disease duration 10 y, working part-time, partnered, MeDi-KD)

“I’ve gone to social things and I just can’t eat anything. So I just sit there and have a glass of water,” (K060; female, age 54 y, disease duration 1 y, not working, partnered, MeDi-KD)

“I’m not taking my MCT with me for dinner when I’m going out.” (K008; male, age 71 y, disease duration 10 y, not working, partnered, MeDi-MCT)

##### Subjective norms, subtheme 2: Professional guidance from registered dietitian

The encouragement, expertise and accountability offered by the KIM trial’s registered dietitian (YW) provided an important counterbalance to outside social pressures discouraging adherence:

“[YNW’s] interventions on a weekly basis were very encouraging, very motivating, kept me going. You know, I wasn’t as good a client or whatever as I could be. And [she] would say, try this other thing. That was important…Knowing that [she was] going to be calling me, I think it would help keep the focus.” (K022; male, age 80 y, disease duration 10 y, working part-time, partnered, MeDi-KD)

“We had a lot of questions about the recipes, but we had a very good relationship with [YNW]…which was very helpful” (K067; male, age 81 y, disease duration 8 y, not working, partnered, MeDi-KD)

## Discussion

Understanding the factors affecting dietary intervention adherence is essential both to the design of future trials and to the eventual clinical translation of regimens found to provide benefit [42]. The purpose of this secondary analysis of the KIM trial was to characterize the barriers and facilitators of adherence to 2 Mediterranean-ketogenic interventions speculated to confer health benefits in PwPs. Reflexive thematic analysis applied to transcripts of TPB-guided semistructured interviews revealed several personal, disease-related, and situational factors impacting adherence to both a low-carbohydrate, high-fat (KD) and MCT-supplemented MeDi variations. Individual attitudes and control beliefs strongly impacted initial behavioral uptake of the study diets, whereas perceptions of familial, community, and professional social support—encompassed by the “Subjective norms” TPB domain—appeared essential to maintenance of dietary adherence. Attitudes toward the compositions and expected health implications of study diet adherence diverged based on past dietary habits and preferences, preformed health and nutrition beliefs, as well as emergent symptoms and dietary experiences over the course of the trial. Self-assessed capacity to meet the physical, attentional, psychological and resource demands of the dietary programs varied based on interactions between personal, disease, and demographic characteristics and was generally lower during the MeDi-KD than the MeDi-MCT phase. Ultimately, however, sustained adherence throughout the intervention phases most often hinged upon a conducive social environment, with tangible care partner support being foundational for many participants and professional dietetic coaching guiding others in surmounting perceived cultural and interpersonal barriers within home and community settings.

There was a high degree of concordance between factors affecting dietary adherence in our cohort and those identified in a systematic review of 12 observational and 6 qualitative studies of Mediterranean-style dietary adherence in adults [64], namely: nutritional attitudes and beliefs, perceived health and weight loss benefits of the diets, diet palatability, familiarity and variety, motivation to change existing dietary habits, self-efficacy, time-intensiveness of food preparation, food access and cost, nutrition knowledge and cooking skills, and perceived social support for dietary adherence within and outside the home. Common barriers pertaining to MeDi-specific elements in both studies were: *1*) incongruities with other traditional cuisines, *2*) weight concerns related to high dietary olive oil content, and *3*) limited experience incorporating pulses and legumes into everyday meals [64]. In the KIM trial, the MeDi-KD exacerbated some of these barriers due to its even greater emphasis on olive oil than is typical of traditional MeDi varieties, and the uniquely high cost burden associated with premium low-carbohydrate food products [65]. These considerations, coupled with the MeDi-KD’s restrictiveness, exacting food preparation requirements, and consequent interference in everyday social and occupational activities, made the MeDi-MCT the more acceptable diet to our cohort in aggregate [43].

### Attitudes

A subset of participants experienced extreme distaste for and/or intolerable gastrointestinal side effects from the MCT oil, resulting in early lapses in adherence to or withdrawal from the MeDi-MCT phase. Our findings thus reaffirm the need for heightened vigilance toward gastrointestinal side effects evoked by any substantive dietary change in PwP, given the high prevalence and heterogeneity of gastrointestinal dysfunction in this population [66]. As articulated by one of our KIM trial participants at the time of withdrawal, the implications of changes in gastrointestinal motility for dopaminergic medication pharmacokinetics—and thus motor, in addition to nonmotor, function—makes this consideration all the more salient [67].

Gastrointestinal effects may be foremost, but are far from singular, among several disease-specific concerns to be heeded when selecting between and personalizing various dietary interventions for PwP. For example, the involved nature of food preparation in the MeDi-KD may make this program less suitable for participants with complex medication schedules, apathy, cognitive slowing, or severe motor symptoms interfering with kitchen tasks [33,38]. PD-associated changes in taste and smell perception, may have wide-ranging effects on flavor preferences and tolerances (i.e., for MCT oil, olive oil, or non–oil-based alternatives) [68,69], which registered dietitians can work collaboratively with PwPs to disentangle.

In addition to personalized meal plan selection and tailoring based on taste preferences and tolerability, our data suggest that dietitians can increase dietary regimen uptake through education around their health benefits and correction of underlying nutrition misconceptions. During the MeDi-KD phase of the KIM trial, adherence may have been augmented with enhanced education around the distinct health profiles of unsaturated and saturated fats. Likewise, underappreciation of pulse/legume consumption as a core MeDi component may account for some of the discordance between our cohort’s high overall baseline self-assessments of MeDi adherence with low mean baseline scores on the MEDAS [62] and may have been corrected for during the MeDi-MCT through appropriate counseling. Increasing pulse and legume intake has been suggested as a promising health-promotion strategy for the general adult population [70] and may prove especially valuable for PwPs, given their demonstrated capacity to address common gastrointestinal (i.e., constipation) complaints [28], associations with reduced PD risk [13,[71], [72], [73]] and putative neuroprotective effects related to high antioxidant contents [73,74].

### Perceived behavioral control

Coaching around versatile, convenient methods of preparing legume and pulse-based dishes may simultaneously address some PwPs’ self-professed deficits in cooking capacity. These low-cost, low glycemic index, micronutrient-rich sources of high-quality protein, carbohydrates, and fiber can partially substitute for pricier MeDi components, such as fish and fresh produce, thereby facilitating MeDi adoption across socioeconomic strata [75,76].

As posited by the TPB, efforts to shape participant attitudes, enhance skills, and best leverage available resources may be most effective when paired with self-efficacy–targeted interventions, such as the self-monitoring tasks deployed in the KIM trial [[77], [78], [79], [80], [81], [82], [83]]. Importantly, however, our findings highlight how thoughtful selection of and education around self-monitoring tasks is critical to guard against paradoxical demoralization attributable to their misinterpretation. This phenomenon appears to be particularly true for biofeedback tasks [32,84,85], as was the case for multiple participants who were discouraged by underwhelming changes in at-home blood ketone measurements. Self-monitoring in the form of intermittent food-related behavior tracking—applicable across an array of dietary interventions, low-burden, and independent of the variability inherent in physiologic responses to food intake—may therefore be preferable to biofeedback from ketone level self-monitoring in a community setting, with the distinct benefit of increasing dietary mindfulness.

### Subjective norms

Effective as these individually-targeted approaches may be in facilitating dietary uptake, our data and others’ suggest that sustained adherence requires thoughtful engagement with the social contexts in which individuals’ food selection, preparation, and eating behaviors take place [64,86]. Dietitian-led couples- and family-based interventions [[87], [88], [89]], combined with communication and contingency planning around meals eaten outside the home [90], may most effectively address subjective norms interfering with adherence in multiple settings. Cultural factors influencing diet acceptability in familial and community realms should likewise be taken into account in the design of such interventions [64,[91], [92], [93], [94], [95]]. For instance, several KIM trial participants’ comments mirror previously identified challenges implementing low-carbohydrate KDs among those accustomed to traditional Asian diets, many of which are characteristically lower-fat and higher-carbohydrate than Western eating patterns [[91], [92], [93]], centering around starchy food staples like rice [96]. For families accustomed to these cultural cuisines, MeDi variants emphasizing the common cross-cultural pairing of local whole grain and legumes/pulse sources—with (i.e., the MeDi-MCT) or without additional ketone supplementation—may represent a more acceptable regimen than carbohydrate-restricted MeDis (i.e., the MeDi-KD) [64,95,[97], [98], [99]].

It is indeed imperative to note that where standard MeDis can and are readily recommended for PwPs in current dietetics practice, based on the wealth of evidence for the overall health benefits [100], phase III studies are still needed to establish any definitive clinical benefit of dietary or supplement-based ketogenic modifications of this pattern in PwPs [101,102]. Understandably, our participants expressed that their future intentions to follow either version of a keto-adapted MeDi would depend on a balance between the feasibility considerations explored in this study and the accrual of more convincing efficacy data in the literature. Future trials could consider comparing alternatives to MCT oil, such as ketone monoesters, to standard MeDis, given their greater capacity to induce nutritional ketosis [[103], [104], [105], [106]], superior gastrointestinal tolerability profile [107], and potential to circumvent the KD-associated reductions in endurance exercise capacity noted by several of our participants [[108], [109], [110]].

### Limitations

Several limitations of the qualitative analysis presented herein need to be taken into account when using our data to inform the design of future trials. Although all KIM trial participants were given the opportunity to complete semistructured interviews, several elected not to do so, particularly when withdrawing from an intervention. In most cases, declined interviews appear to have been attributable to logistical concerns (i.e., time constraints); however, we cannot rule out the possibility that feedback from these participants would have differed systematically from that collected by agreeable interviewees. In keeping with the demographics of the full study cohort, interviewees were distributed across a relatively narrow distribution of age, disease severity and duration, in part due to our study criteria excluding individuals with advanced PD (Hoehn &Yahr stage IV). Our analysis thus did not substantively address the unique nutritional concerns for PwPs with later-stage disease, such as chewing and swallowing difficulties, appetite and weight loss, and gastrointestinal disturbances associated with LCIG [111]. Indeed, whereas weight loss was a stated goal for many of our participants, weight loss is understood as a health risk for those with advanced PD [[112], [113], [114], [115]], which may render the MeDi-KD unsuitable for this subpopulation. Underrepresentation of women in our sample relative to the broader population of PwPs may have led us to under-detect challenges related to food preparation, as women in our study and previous studies of PwPs and older adults are less likely to receive support from spouses in managing their disease and household responsibilities alike [[116], [117], [118]]. Although several of our interviewees provided rich constructive feedback regarding cultural impediments to study diet adherence, the underrepresentation and relative homogeneity of ethnic minorities in our study means our data undoubtedly fails to fully capture the breadth and prevalence of such barriers extrapolated to the general population of PwPs. Likewise, our cohort was predominantly highly educated, having completed ≥1 postsecondary degree, and lived in a high socioeconomic status urban area in close proximity to the academic center at which the study was performed. As such, the true implications of diet-associated costs and food access issues in more remote locations are likely underestimated by direct extrapolation of our findings. Directed efforts to recruit a more demographically diverse cohort are warranted in future studies of dietary interventions in PD, for which various inventive approaches have been suggested [[119], [120], [121], [122]]. Finally, volunteers for dietary intervention trials are well-documented to score higher in health consciousness and trait self-efficacy than the broader population [123], meaning that behavioral uptake and maintenance of dietary adherence documented in the KIM trial feasibility results [43] can be presumed to overestimate what would be seen in real-world practice.

### Conclusion

This study offers valuable insights into the barriers and facilitators of adherence to 2 Mediterranean-ketogenic dietary interventions in PwPs. By structuring our analysis within the TPB, we systematically disentangled the relative importance of attitudinal, normative, and control-related factors in determining dietary uptake and maintenance. In clarifying mechanisms by which personal and disease characteristics interacted with home and community supports to influence adherence, our study offers insights for future interventions involving this population, including strengthening self-efficacy through low-burden self-monitoring practices, modifying food preparation instructions to accommodate motor and attentional limitations, and engaging family members in culturally-informed nutritional counseling sessions [42,64,76].

Importantly, our findings extend beyond the design of future trials to offer insights directly relevant to clinical care. Although the efficacy of keto-adapted MeDi interventions in PD remains to be established, demand for general dietary counseling among PwPs is increasing with the growing recognition of the importance of nutrition in disease management [124]. The results of our analysis suggest that dietary guidance for PwPs should be individualized, accounting for motor and nonmotor symptom burden, self-efficacy levels, social and cultural environments, and economic resources. For many PwPs, particularly those without access to professional dietetic support, modified MeDi approaches combined with ketone supplementation may prove more practical and sustainable than carbohydrate-restricted KD variants, although tolerability must be carefully assessed. Clinicians should also be aware of the potential for both encouraging and discouraging effects of biofeedback on adherence, and consider emphasizing flexible, food-focused monitoring strategies over stringent tracking of ketone levels in community settings.

## Author contributions

The authors’ responsibilities were as follows—TRC, SA-C: designed research; KNT, YNW: conducted research; KNT: analyzed data and performed statistical analysis; KNT, YNW, JSTL, TRC, SA-C: contributed to the writing of the paper; SA-C: had primary responsibility for final content; and all authors: read and approved the final manuscript.

## Data availability

Data described in the manuscript, code book, and analytic code will not be made available given the risk of deidentifying participants through the sharing of interview audio recordings or transcripts.

## Funding

The trial was funded by the Weston Family Foundation. KNT is funded by the Canada Graduate Scholarships Doctoral (CGS-D) and UBC MD/PhD program. YNW is funded by the Canada Graduate Scholarships Master’s (CGS-M) program. JSTL is funded by a Parkinson Canada Graduate Student Award.

## Conflict of interest

SA-C is supported by the Marg Meikle Professorship for Parkinson’s Research and has received grant funding from the Pacific Parkinson’s Research Institute, the Weston Family Foundation, Parkinson Canada, Parkinson Society British Columbia, Rick’s Heart Foundation, Canadian Institutes of Health Research, Jack and Darlene Poole Foundation, VGH and UBC Hospital Foundation, and UBC Research Excellence Clusters program. SA-C is the site principal investigator for industry-funded clinical trials into essential tremor by AbbVie and Praxis Precision Medicine, and has received honoraria for teaching from Merz and AbbVie and consulting fees from Sunovion, AbbVie, Ipsen, and Merz. All other authors report no conflicts of interest.
