# Supplementary material for: Exploring the Facilitators and Barriers of Adherence to Mediterranean-Ketogenic Dietary Interventions in Parkinson’s Disease: A Qualitative Study
Source: Curr Dev Nutr. 2025 Oct 30;9(11):107591. doi: 10.1016/j.cdnut.2025.107591 (PMC12670100; doi:10.1016/j.cdnut.2025.107591)
Supplement: multimedia component 1 [file mmc1.docx]

**Supplementary Table 1. Individual Participant Characteristics.**

| **ID** | **Sex** | **Age** | **Ethnicity** | **HY** | **Disease Duration (Yrs)** | **Employment** | **Relationship Status** | **Interventions Completed** | **Interventions Withdrawn** |
| --- | --- | --- | --- | --- | --- | --- | --- | --- | --- |
| K002 | M | 81 | Caucasian | II | 10 | Not working | Partnered |  | MeDi-KD |
| K004 | M | 74 | Caucasian | II | 4 | Not working | Partnered | MeDi-KD & MeDi-MCT |  |
| K005 | F | 63 | Caucasian | I | 22 | Not working | Partnered | MeDi-KD & MeDi-MCT |  |
| K006 | F | 68 | Caucasian | II | 8 | Not working | Single |  | MeDi-MCT |
| K007 | M | 66 | East Asian | II | 9 | Not working | Partnered | MeDi-KD & MeDi-MCT |  |
| K008 | M | 71 | Caucasian | II | 10 | Not working | Partnered | MeDi-KD & MeDi-MCT |  |
| K009 | F | 76 | Caucasian | II | 6 | Not working | Partnered | MeDi-KD & MeDi-MCT |  |
| K011 | M | 74 | Caucasian | II | 11 | Not working | Single | MeDi-KD & MeDi-MCT |  |
| K014 | M | 63 | Caucasian | II | 6 | Not working | Single | MeDi-MCT | MeDi-KD |
| K015 | M | 74 | Caucasian | II | 13 | Not working | Partnered | MeDi-KD & MeDi-MCT |  |
| K016 | M | 59 | Caucasian | II | 13 | Not working | Single |  | MeDi-KD |
| K017 | F | 69 | Caucasian | I | 2 | Not working | Partnered | MeDi-KD & MeDi-MCT |  |
| K019 | F | 75 | Caucasian | II | 12 | Not working | Single | MeDi-KD & MeDi-MCT |  |
| K020 | M | 76 | Caucasian | II | 5 | Not working | Single | MeDi-KD & MeDi-MCT |  |
| K022 | M | 80 | Caucasian | II | 10 | Working part-time | Partnered | MeDi-KD & MeDi-MCT |  |
| K024 | M | 69 | Caucasian | II | 10 | Working part-time | Partnered | MeDi-MCT | MeDi-KD |
| K025 | M | 50 | East Asian | II | 7 | Working part-time | Partnered | MeDi-KD & MeDi-MCT |  |
| K026 | M | 56 | Caucasian | II | 9 | Not working | Single | MeDi-KD & MeDi-MCT |  |
| K027 | M | 70 | Caucasian | II | 8 | Not working | Partnered | MeDi-KD & MeDi-MCT |  |
| K031 | F | 65 | Caucasian | I | 13 | Not working | Partnered | MeDi-KD & MeDi-MCT |  |
| K032 | M | 67 | Caucasian | II | 12 | Not working | Partnered | MeDi-KD & MeDi-MCT |  |
| K033 | F | 64 | East Asian | I | 7 | Not working | Partnered | MeDi-KD & MeDi-MCT |  |
| K034 | M | 57 | Caucasian | III | 2 | Not working | Partnered | MeDi-KD & MeDi-MCT |  |
| K035 | M | 72 | South Asian | II | 6 | Working part-time | Partnered | MeDi-KD & MeDi-MCT |  |
| K038 | F | 75 | Caucasian | II | 2 | Not working | Partnered | MeDi-KD & MeDi-MCT |  |
| K039 | M | 68 | Caucasian | II | 3 | Not working | Partnered | MeDi-KD & MeDi-MCT |  |
| K041 | M | 57 | Caucasian | II | 13 | Working part-time | Single | MeDi-KD & MeDi-MCT |  |
| K045 | M | 59 | Caucasian | II | 2 | Working full-time | Partnered | MeDi-KD | MeDi-MCT |
| K046 | F | 63 | Caucasian | I | 5 | Not working | Single | MeDi-KD | MeDi-MCT |
| K048 | M | 56 | East Asian | II | 19 | Working part-time | Partnered | MeDi-KD & MeDi-MCT |  |
| K049 | M | 60 | Caucasian | II | <1 | Working part-time | Partnered | MeDi-KD & MeDi-MCT |  |
| K050 | F | 68 | Caucasian | II | 8 | Not working | Partnered | MeDi-MCT | washout following completion of MeDi-MCT |
| K051 | M | 68 | Caucasian | II | 8 | Not working | Partnered | MeDi-KD & MeDi-MCT |  |
| K053 | F | 56 | Caucasian | I | 10 | Not working | Partnered | MeDi-KD & MeDi-MCT |  |
| K055 | M | 79 | Caucasian | II | 5 | Not working | Partnered | MeDi-KD & MeDi-MCT |  |
| K056 | F | 64 | Caucasian | II | 12 | Not working | Partnered | MeDi-KD | washout following completion of MeDi-KD |
| K057 | M | 62 | Caucasian | III | 16 | Not working | Partnered | MeDi-KD & MeDi-MCT |  |
| K059 | M | 74 | Caucasian | I | 3 | Not working | Partnered | MeDi-KD & MeDi-MCT |  |
| K060 | F | 54 | Caucasian | II | 1 | Not working | Partnered | MeDi-KD & MeDi-MCT |  |
| K062 | M | 77 | Caucasian | II | 9 | Not working | Partnered | MeDi-MCT | washout following completion of MeDi-MCT |
| K063 | M | 61 | Caucasian | III | 4 | Working full-time | Single | MeDi-KD | washout following completion of MeDi-KD |
| K066 | M | 78 | Caucasian | II | 4 | Not working | Partnered | MeDi-KD & MeDi-MCT |  |
| K067 | M | 81 | Caucasian | II | 8 | Not working | Partnered | MeDi-KD & MeDi-MCT |  |
| K068 | M | 71 | Caucasian | II | 3 | Not working | Partnered | MeDi-KD & MeDi-MCT |  |

**Supplementary Table 2. Percentage of interviews with references to individual codes.**

| **Codes** | **Intervention = MeDi-KD (n=32)** | **Intervention = MeDi-MCT (n=35)** |
| --- | --- | --- |
| **Challenge adapting to unfamiliar foods** | 19% | 14% |
| **Familiarity with study diet** | 25% | 31% |
| **Positive attitude towards dietary change** | 28% | 14% |
| **Meal plans or MCT oil were unpalatable** | 19% | 26% |
| **Meal plans were palatable** | 38% | 37% |
| **Diet perceived as flexible** | 19% | 23% |
| **Diet perceived as too restrictive** | 50% | 29% |
| **Diet perceived as generally healthy** | 47% | 26% |
| **Diet perceived as generally unhealthy** | 28% | 9% |
| **Experienced adverse side effects** | 38% | 40% |
| **Experienced symptom improvement** | 69% | 40% |
| **Lack of experienced symptomatic benefits** | 9% | 14% |
| **Perceived lack of evidence for health benefit** | 6% | 23% |
| **High trait self-efficacy** | 22% | 14% |
| **Food diaries increased adherence self-efficacy** | 13% | 3% |
| **Food diaries were onerous** | 3% | 6% |
| **Ketone self-monitoring reinforced efforts** | 28% | 14% |
| **Ketone self-monitoring was technically challenging** | 13% | 29% |
| **Low ketone readings discouraged efforts** | 9% | 11% |
| **Inadequate food preparation skills** | 9% | 9% |
| **Versatile cooking skills** | 9% | 3% |
| **Recipe ingredients were hard to find** | 28% | 23% |
| **Recipe ingredients were readily accessible** | 6% | 6% |
| **Grocery lists were more expensive than usual** | 34% | 17% |
| **Competing disease management tasks** | 3% | 11% |
| **MCT doses were difficult to integrate into routine** | 0% | 26% |
| **MCT doses were easy to incorporate into routine** | 0% | 49% |
| **Time-intensive food preparation** | 66% | 23% |
| **Diets viewed as incompatible with eating norms outside the home** | 50% | 34% |
| **Diets were incompatible with household or community cultural cuisine** | 9% | 11% |
| **Tangible support and encouragement from care partner** | 19% | 14% |
| **Information, implementation strategies and encouragement from RD** | 22% | 20% |
